# Experimental and Numerical Study on Effect of Sample Orientation on Auto-Ignition and Piloted Ignition of Poly(methyl methacrylate)

**DOI:** 10.3390/ma8074004

**Published:** 2015-07-02

**Authors:** Fei Peng, Xiao-Dong Zhou, Kun Zhao, Zhi-Bo Wu, Li-Zhong Yang

**Affiliations:** 1 State Key Laboratory of Fire Science, University of Science and Technology of China, Hefei 230026, Anhui, China; E-Mails: pfei@mail.ustc.edu.cn (F.P.); zxd@ustc.edu.cn (X.-D.Z.); zk008@mail.ustc.edu.cn (K.Z.); wuzhibo@mail.ustc.edu.cn (Z.-B.W.); 2 Collaborative Innovation Center for Urban Public Safety, 96 Jinzhai Road, Hefei 230026, Anhui, China

**Keywords:** orientation, piloted ignition, auto-ignition, critical mass flux

## Abstract

In this work, the effect of seven different sample orientations from 0° to 90° on pilot and non-pilot ignition of PMMA (poly(methyl methacrylate)) exposed to radiation has been studied with experimental and numerical methods. Some new and significant conclusions are drawn from the study, including a U-shape curve of ignition time and critical mass flux as sample angle increases for pilot ignition conditions. However, in auto-ignition, the ignition time and critical mass flux increases with sample angle α. Furthermore, a computational fluid dynamic model have been built based on the Fire Dynamics Simulator (FDS6) code to investigate the mechanisms controlling the dependence on sample orientation of the ignition of PMMA under external radiant heating. The results of theoretical analysis and modeling results indicate the decrease of total incident heat flux at sample surface plays the dominant role during the ignition processes of auto-ignition, but the volatiles gas flow has greater influence for piloted ignition conditions.

## 1. Introduction

Poly(methyl methacrylate) is widely used as decorative and construction materials in factories, hospitals, traffic tools and so on. However, high heating value, heavy smoke, and severe toxicity of poly(methyl methacrylate) will cause serious hazards for life and properties once fire occurs in buildings [1]. When exposed to a high enough incident heat flux, poly(methyl methacrylate) begins to decompose and produce volatile species, the volatile gas then mixes with the ambient air to produce a flammable mixture; when the concentration of combustible gas rises up to the lower flammability limit and temperature is high enough or a pilot exists, ignition occurs. The subject of ignition is fundamental to combustion science and of great practical importance. It is the primary step in the whole fire strategies and plays a critical role in fire growth; understanding the mechanism of ignition is very important for predicting the occurring of fire and for designing fire protecting standard.

Many researchers studied the ignition process of solid fuels [2,3,4,5,6,7]. Two types of ignition are possible: auto-ignition and piloted ignition [8]. Bilbao [9] studied the effect of the two types of ignition and the distance between the heating source and the sample, longer ignition time are observed for auto-ignition than for piloted ignition. Wu [10] studied the effect of pilot location and energy through experimental methods. However, there are few researches about the effect of orientation on ignition of solid fuels. Babrauskas [11] reviewed the effect of sample orientation on ignition from experiments, which concluded the ignition time of vertical sample is longer. Babrauskas [11], Shields [4] and Tsai [12] found the ignition time ratio of vertical and horizontal is 1.2/1.4. However, with the different location of pilot, Yang *et al.* [13] found ignition times of wood for vertical orientation are shorter than horizontal, which is different from the result of Babrauskas and Shields. Tsai [12] conducted experimental work on the orientation effect to study the thermal processes in solid prior to ignition, while the pyrolysis process was ignored. Gotoda and Yuji *et al.* [14,15] used a CO_2_ laser as an external radiant source, studied the sample orientation effect (from −90° to 90°) of thermally thin (0.2~0.5 mm) poly(methyl methacrylate) at very high heat flux (1200 to 2050 kW/m^2^). Chen *et al.* [16] studied the upward, downward, and vertical cases of piloted ignition using thick paulownia wood, and found that the shortest ignition time turns out to be for downward orientation, but the longest ignition time for vertical orientation. However, it is evident that just few researchers studied the materials exposed to the orientation, which are not just multiples of 90° and there are few reports about auto-ignition behavior under different orientations. 

The present article has investigated the influence of sample orientation with comparative experiments, in which seven orientations have been employed, and both the piloted and auto-ignition situations are considered. Furthermore, a computational fluid dynamic model have been built based on the Fire Dynamics Simulator (FDS6) to investigate the mechanisms control the dependence on sample orientation of the ignition of PMMA under external radiant heating.

## 2. Experimental Details

### 2.1. Apparatus Methods

**Figure 1:**
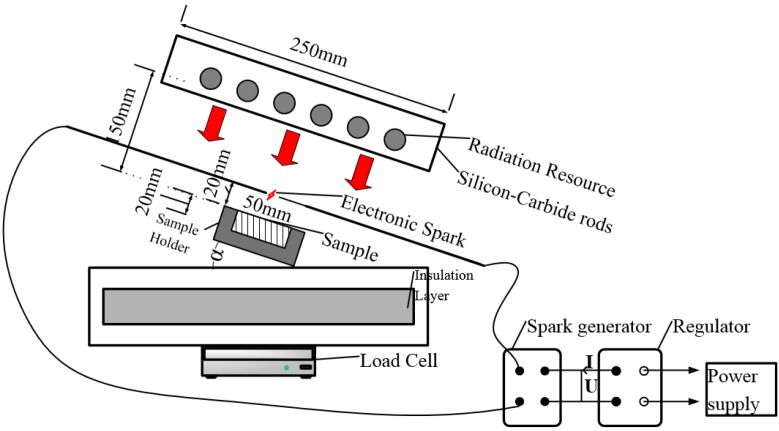
Schematics of experimental apparatus.

All experiments are conducted using a movable radiation platform, which is developed to investigate the pyrolysis and ignition behavior of solid materials exposed to a radiation. As shown in Figure 1, the experimental apparatus, in which the orientation of sample can be adjusted, consists of the radiation source, load cell, water cooled heat flux meter, electric spark igniter and data acquisition system. The radiation source, which is made of six silicon–carbide bars, can supply a uniform radiant heat flux of 0–45 kW/m^2^ at the test sample surface, and the size of heating area is 250 mm × 250 mm. The incident heat flux is measured using the heat flux meter before each test.

Poly(methyl methacrylate) cut into a size of 50 mm × 50 mm × 20 mm is used as the experimental samples. In order to ensure insulation conditions on the sides, all the sides of the samples are wrapped in asbestos (as asbestos is harmful to human health, other thermal insulation materials are recommended to replace asbestos) with an aluminum foil outermost layer except the upper surface of 50 mm × 50 mm is exposed to radiation. An electronic balance is used as a load cell to record the mass loss of the sample during thermal decomposition.

**Figure 2:**
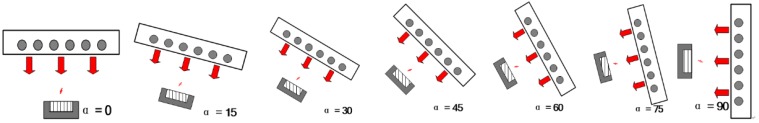
Schematic of orientation change (changes every 15°).

In the experiments, the radiation source is placed at seven different orientations (namely α = 0°, 15°, 30°, 45°, 60°, 75°, 90°) to supply the heat flux for samples as shown in Figure 2. For each orientation, the sample and radiation source is changed at the same time to keep the sample surface parallel to the radiation source. The distance between radiation source and sample is held at approximately 150 mm, regardless of any sample orientation. All experiments are repeated more than four times to ensure accuracy and reliability.

Both piloted and auto-ignition is presented in the study. The piloted ignition tests is induced with a continuous spark, which is widely used in present experiments, placed 20 mm above sample middle surface. In the non-piloted situation, the sample exposed to the thermal radiation without the spark until ignite occurs.

### 2.2. Procedure and Observed Fire Behavior

First of all, radiation source adjusted to an expected orientation. Then turn on the radiation source and adjust to the expected heat flux level, following which the data acquisition system is turned on. The heat source needs a few minutes to achieve a stable status. When the heat flux measured becomes steady, the sample is put under the heat source and the experiment begins.

**Figure 3:**
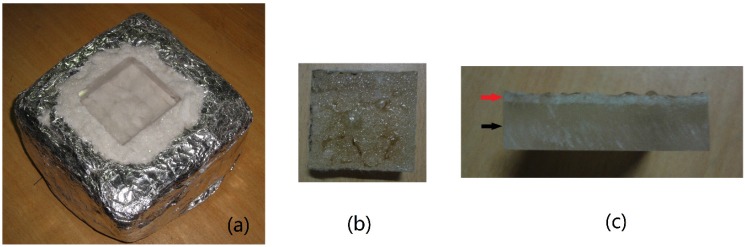
Poly(methyl methacrylate) (PMMA) sample: (**a**) Before experiment; (**b**) Front face after experiment; (**c**) Side face after experiment.

When exposed to the radiation heat flux, the sample begins to receive heat. In a few seconds, the first layer melts and begins to boil, as shown in Figure 3. Meanwhile, PMMA decomposes and volatile species is released. The volatile gases layer become thicker as time goes on. After a period of time, a flame occurs above the sample surface. 

When ignition occurs, one experiment can be ended. However, if the sample does not ignite after being exposed to radiation in 15 minutes, it will be judged that the sample cannot ignite under that condition. Figure 3 shows the PMMA sample before and after the experiment.

## 3. Numerical Modeling

In order to investigate the parameters that influence the pyrolysis and ignition process, a numerical model based on the Fire Dynamics Simulator is built. Using the numerical model, the parameters of temperature field, concentration field, heat release field, heat flux at sample surface can be gained.

The numerical model is a two-dimensional simulation of the piloted ignition experiments carried out on the movable radiation platform of PMMA. The model is developed using the Fire Dynamics Simulator (FDS 6.0.2) in its Direct Numerical Simulation mode considering simultaneously the processes both in the solid phase and gas phase. The pyrolysis process in solid phase is modelled using a single step global Arrhenius reaction: −[CH_2_C(CH_3_)(COOCH_3_)]_n_− → nCH_2_C(CH_3_)(COOCH_3_). In addition, oxidative pyrolysis has not taken into consideration. A single-step second order Arrhenius reaction is used to model the gas phase finite rate chemical reaction: C_5_H_8_O_2_ + 6O_2_ → 5CO_2_ + 4H_2_O.

**Figure 4:**
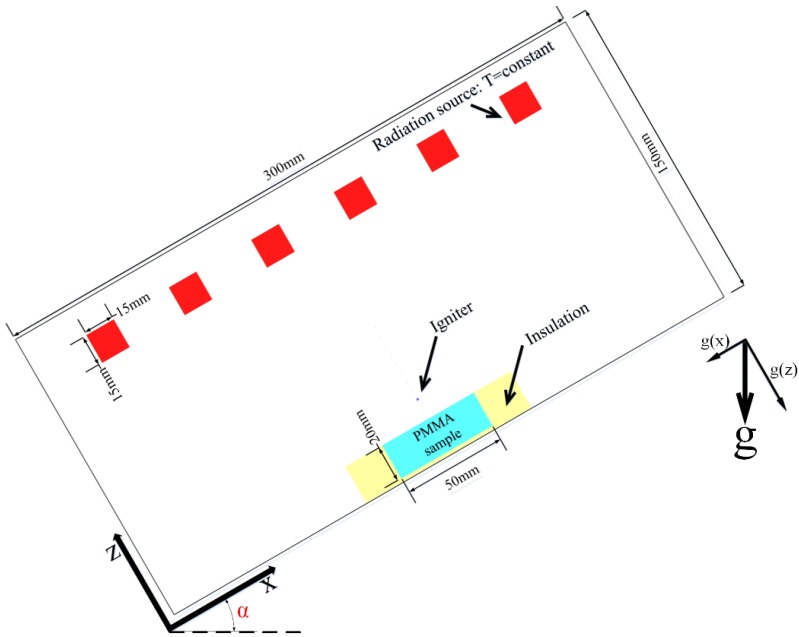
Schematic of computational domain.

As shown in Figure 3, the computational domain is a size of 300mm in x direction and 150mm in z direction. The cell size of the computational domain is 1 mm × 1 mm for both solid- and gas-phase in all simulations. The radiation source is modelled as six constant-temperature blackbody surface blocks of the size 15 mm × 15 mm placed 15cm above the sample surface. In this study, for piloted ignition conditions, the igniter is modelled as a single grid region at a constant temperature of 1200 °C placed 20 mm above the sample middle surface. The igniter is removed for auto-ignition conditions. The emissivity of the igniter is kept at 0.001 to minimize its influence on radiant heating the sample surface. The orientation of gravitational acceleration is changed to simulate the change of sample orientation, the orientations of g, g(x) and g(z) are shown in Figure 4. The values of g(x) and g(z) are shown in Table 1, where g = −9.81 m/s^2^; g(x) = *g* cos(*α*) and g(z) = *g* sin(*α*).

**Table 1:** Values of *g*(x) and *g*(z) at different orientation. (Unit: m/s^2^)

Orientation	0°	15°	30°	45°	60°	75°	90°
g(x)	0	−2.539	−4.905	−6.937	−8.496	−9.476	−9.81
g(z)	−9.81	−9.476	−8.496	−6.937	−4.905	−2.539	0

**Figure 5:**
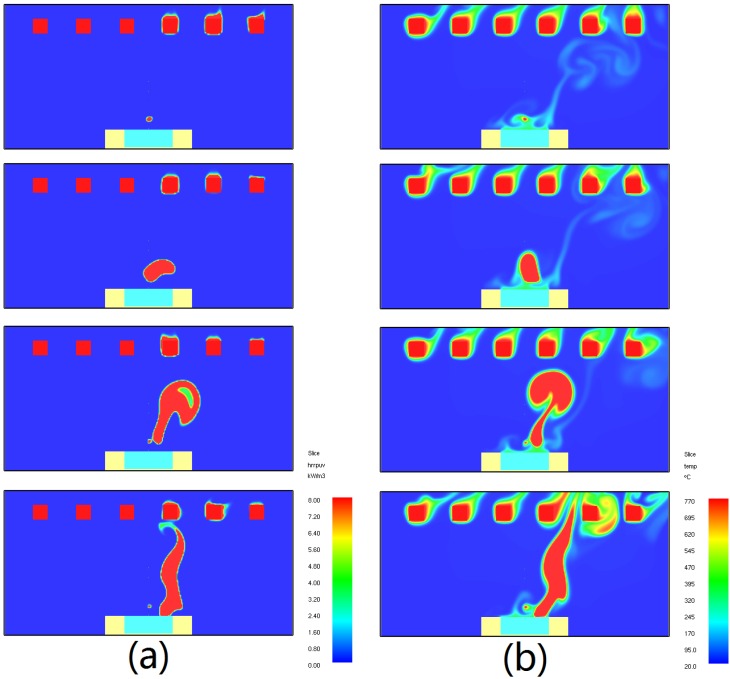
Model results: temperature and heat release rate changes at ignition, (**a**) Heat release rate at ignition when α = 15°; (**b**) Temperature at ignition when α = 30°.

The two rank figures in Figure 5 show the piloted ignition simulation results of temperature changes (Figure 5a) and heat release rate changes (Figure 5b) at ignition time. In order to limit the length of this article, only the results of temperature for 30° and HRR (heat release rate) for 15° are shown, for an example as how to explain how fire occurs in gas and spread from gas phase to solid surface. The progress of ignition is clear, first the combustible gas ignites at the pilot in the gas phase, and then the flame spreads from gas phase to solid surface very rapidly (within 0.5 s). By observing the sudden changes of temperature and heat release rate in gas, the occurrence of ignition can be easily found.

## 4. Results and Discussion

### 4.1. Ignition Time

The ignition time here is defined as the duration time from when the sample is first exposed to the incident radiant heat flux to the time when a visible flame is observed, which eventually depends on pyrolysis rate and the combustible gas flow.

#### 4.1.1. Piloted Ignition

**Figure 6:**
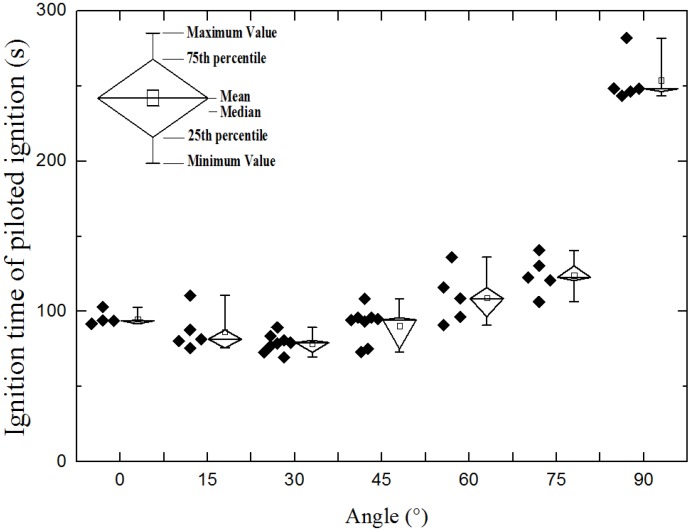
Relationship between ignition time and radiation orientation.

Ignition of solid fuels is investigated by many researchers, and most of the experiments were performed in the piloted ignition mode. Figure 6 shows the piloted ignition experimental results of ignition time at different orientation. There is a U-shape of ignition time can be seen from the figure. The ignition time gets shorter while orientation angle (α) changes from 0° to 30°, but gets longer when α increases from 30° to 90°.

**Figure 7:**
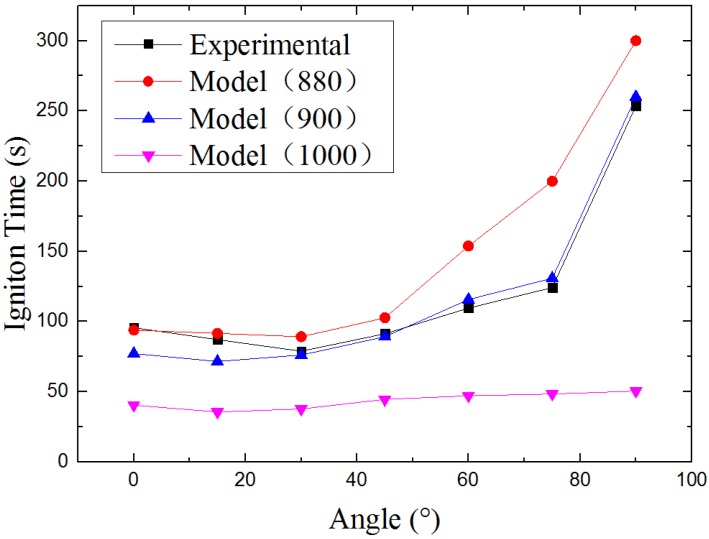
Results: Ignition Time *vs.* Sample Orientation.

The modeling results of ignition time compares with experimental results is shown in Figure 7. Agreeing with the experimental results, under three different radiant heat fluxes, the profile of modeling results has a U-shape. The ignition time gets shorter with the increasing radiation angle when the radiation angle is less than a specific orientation and gets longer when radiation angle is larger than the orientation. The specific orientation angle is α=15° when the surface temperature of heat source is 900 °C and 1000 °C, and α = 30° for a surface temperature of 880 °C. The agreement of experimental and modeling results of U-shape regulation indicates there is some elements (gas flow, heat flux and so on) changes with the sample orientation makes it happen.

#### 4.1.2. Auto-Ignition

In order to ensure the occurrence of auto-ignition, the temperature of the flammable mixture must be sufficiently high to initiate and accelerate the gas-phase exothermic chemical reactions. At any given radiation heat flux and orientation, it is evident that ignition times of auto-ignition will be longer than ignition times of piloted ignition. 

**Figure 8:**
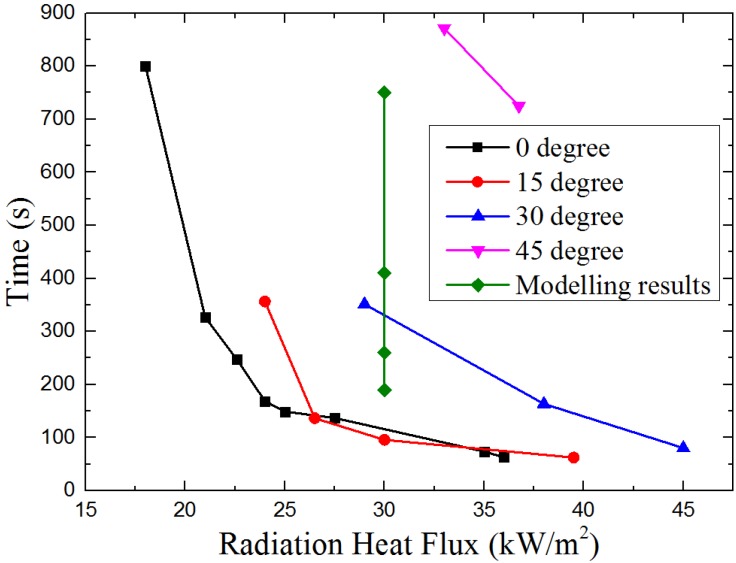
Ignition time *vs.* sample orientation

Figure 8 shows the experimental results and modeling results (the points in green line from bottom to top show ignition time of 0°, 15°, 30° and 45°) of ignition time at different angle of auto-ignition. From the figure we can find that under the same radiant heat flux, ignition time increase from 0° to 45°. Similar results were found by the simulation model. Shown as the green line in Figure 8, under the radiant heat flux of 31 kW/m^2^, ignition time increases from 200 to 700 s when orientation angle increase from 0° to 45°.

The model developed by Delichatsios [17], which has been widely used by other researchers indicates that the inverse of the square root of ignition time is proportional to the radiant heat flux for thermally thick conditions. The relationship of ignition time and radiant heat flux and critical heat flux is given by the formula.

(1) $$\frac{1}{\sqrt{t_{ig}}}=\frac{1}{\sqrt{\pi }}\frac{2{\dot{q}}_{in}^{\prime \prime }+a{\dot{q}}_{cr}^{\prime \prime }}{\sqrt{k\rho c}\left(T_{ig}-T_{0}\right)}$$

**Figure 9:**
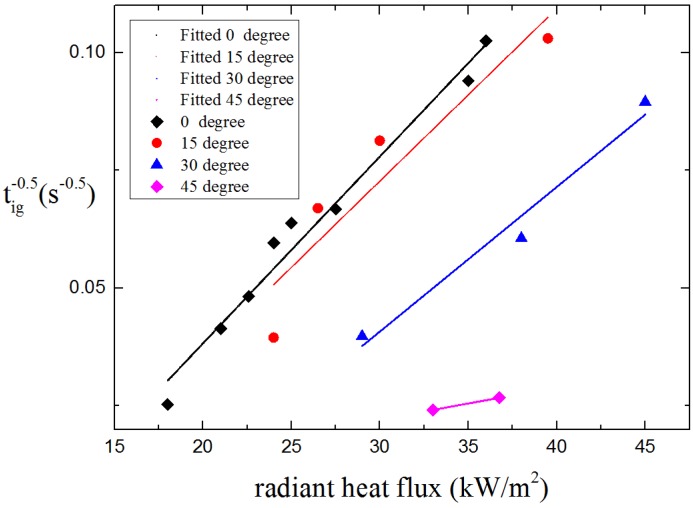
$\sqrt{t_{ig}}$ against radiant heat flux of auto-ignition.

Here, *t_ig_* is the ignition time; *T_ig_* is the ignition temperature; *T*_0_ is the initial temperature; *k* is the thermal conductivity; *ρ* is the density of solid; *c* is the specific heat of sample; ${\dot{q}}_{in}^{\prime \prime }$ is the incident heat flux and ${\dot{q}}_{cr}^{\prime \prime }$ is the critical heat flux for ignition. The measured $\sqrt{t_{ig}}$ and incident heat flux is shown in Figure 9, the correlation between $t_{ig}^{-0.5}$ and ${\dot{q}}_{in}^{\prime \prime }$ is lining fitted as $t_{ig}^{-0.5}=a{\dot{q}}_{in}^{\prime \prime }+b$. Normally ${\dot{q}}_{cr}^{\prime \prime }$ is considered to be the lowest thermal load per unit area (heat flux) capable of ignition, which means when *t_ig_* → ∞ the incident heat flux ${\dot{q}}_{in}^{\prime \prime }$ is equal to ${\dot{q}}_{cr}^{\prime \prime }$, so ${\dot{q}}_{cr}^{\prime \prime }$ can be gained by extrapolation. As shown in Table 2, the critical heat flux also increases from 9.39 to 22.63 kW/m^2^ as orientation angle α increases from 0° to 45°. The result of critical heat flux indicates that it is harder to ignite with the increasing α in auto-ignition conditions. It has to be mentioned that during the experiments when α is larger than 45°, the ignition is very hard to occur under the radiant heat flux of 45 kW/m^2^, so there is only two plots for α = 45° (the errors of lining fitting may bigger than other orientations) and ignition does not occur in 15 min for α > 45°.

**Table 2:** Critical heat flux of auto-ignition.

Orientation	a	b	R-Square	${\dot{q}}_{cr}^{\prime \prime }$
0°	0.00397	−0.3728	0.97382	9.39 kW/m^2^
15°	0.00367	−0.04108	0.90971	11.19 kW/m^2^
30°	0.00307	−0.05143	0.94509	16.75 kW/m^2^
45°	0.000684	−0.0155	---------	22.63 kW/m^2^

### 4.2. Mass Loss Rate

Mass loss rate, which can be used to predict the ignition solid fuels, reflects the process of pyrolysis and the mass flow of combustible gas mix released from solid fuels. The critical mass flux is a physically correct ignition criterion. It has been employed as the ignition critical condition by many researchers. The mass loss rate of both piloted ignition and auto-ignition is investigated by experimental and modeling as follows.

#### 4.2.1. Piloted Ignition

**Figure 10:**
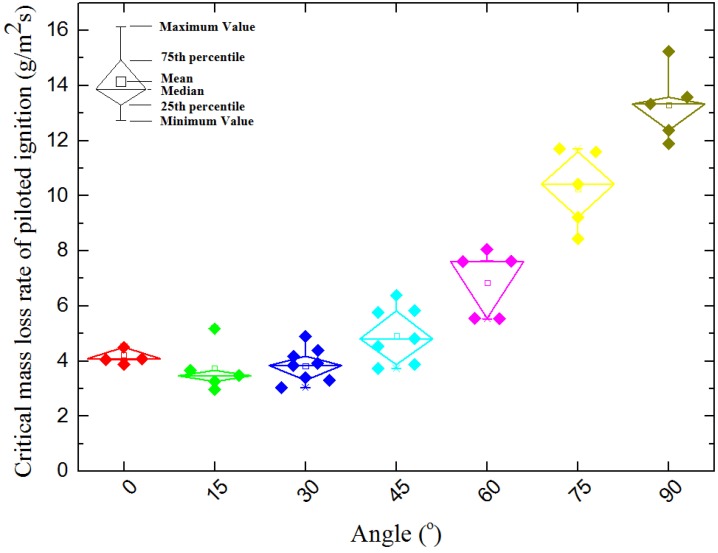
Relationship between critical mass loss rate and orientation.

**Figure 11:**
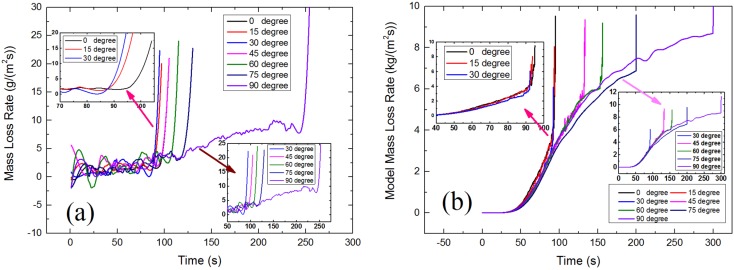
Results of mass loss rate of different radiation orientation: (**a**) Experimental results; (**b**) Modeling results.

Figure 10 shows the critical mass loss rate changes with α. It can be seen that the mass loss rate also has a U-shape relationship with orientation angle. However, the critical mass loss rate basically keeps nearly steady when α increases from 0° to 30°, that indicates sample orientation has relatively less effect on the combustible gas concentration when α is between 0° and 30°(also see the little picture in top left corner of Figure 11a,b). For when α is larger than 30°, the critical mass loss rate increases rapidly (also see the little picture in bottom right corner of Figure 11a,b), so the sample orientation has a greater impact on the combustible gas concentration. Figure 11 shows the experimental and modeling results of mass loss rate of piloted ignition at seven different radiation orientations. For α increases from 0° to 75°, the mass loss rate slightly decreases with α before the occurrence of ignition at all time, this is due to the changes of incident heat flux and surface temperature decrease with α and will be discussed in the next sections.

#### 4.2.2. Auto-Ignition

**Figure 12:**
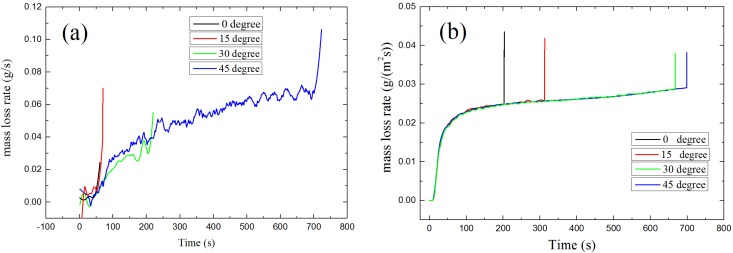
Results of mass loss rate *vs.* time: (**a**) Experimental results; (**b**) Modeling results.

Auto-ignition is a common situation in real fires. Unlike the result of piloted ignition, as shown in Figure 12 the critical mass loss rate of auto-ignition always increase with orientation angle. Without the spark igniter, the auto-ignition of solid fuels require the reaction rate of the flammable mixture to be sufficiently high to produce enough energy to initiate and accelerate the gas-phase exothermic chemical reactions. That means in order to make sure the occurrences of auto-ignition, the concentration of combustible volatiles in gas must be higher, and result in higher critical mass loss rate.

### 4.3. Heat Flux

The incident heat flux at the sample surface has great influence on the pyrolysis and ignition process of solid fuels. Both the influx of radiant heat and out flux of convective heat contributes to the net heat flux at the sample surface.

#### 4.3.1. Radiant Heat Flux

The incident radiant heat flux for heating solid combustible is determined by the heater source. However, it will be selectively absorbed and attenuated by the pyrolysis volatiles in the boundary layer so that the irradiance practically available is substantially less than that without or less absorbing gas [18]. The components of pyrolysis gases have a strong radiation absorption effect in the near-infrared and mid-infrared spectral regions. With the increasing of orientation angle α, the pyrolysis gases layer become thinner and thinner. Therefore, the incident radiant heat flux absorbed by the pyrolysis volatiles is less.

**Figure 13:**
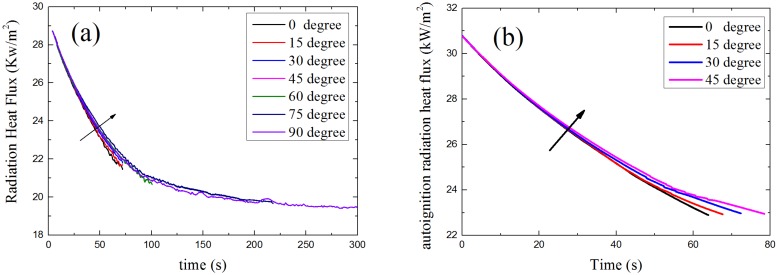
Model results: radiant heat flux *vs.* time: (**a**) Piloted ignition; (**b**) Auto-ignition.

The results of relationship between radiant heat flux and time as α changes from 0° to 90° is given in Figure 13. Both the piloted ignition (Figure 13a) and auto-ignition (Figure 13b) follow the same rule. The radiant heat influx at sample surface decreases with time at all orientation, this because of pyrolysis products concentration increase in gas phase due to radiation attenuation. In addition, also we can see from Figure 11 that the radiant heat flux gets larger with the increasing α. Radiant heat flux is lowest at the orientation angle of 0°. The variation in radiant heat flux with α shows that radiation attenuation deceases with the increasing α.

#### 4.3.2. Convective Heat Flux

The convective heat transfer coefficient and convective heat flux are not the same for different oriented samples. This is one of the reasons why ignition behaviors vary for different oriented samples. Generally, the convective heat transfer coefficient is calculated from the Nusselt number *Nu_L_*, the value of *Nu_L_* is different for different orientations [19].

For α = 0° (upward hot surface),

(2) $$Nu_{L}=0.54Ra_{L}^{1/4}$$

For α = 15°~75° (hot surface facing up), average Nusselt number is,

(3) $$\bar{Nu_{L}}=0.14[{\left(Gr_{L}Pr\right)}^{1/3}-{\left(Gr_{C}Pr\right)}^{1/3}]+0.56{\left(Ra_{L}\cos\theta \right)}^{1/4}$$

The value of *Gr_C_* in Equation (3) is listed as below:

α = 15°, *Gr_C_* = 5 × 10^9^; α = 30°, *Gr_C_* = 2 × 10^9^; α = 45°, *Gr_C_* = 10^9^; α = 60°, *Gr_C_* = 10^8^; α = 75°, *Gr_C_* = 10^5^.

For α = 90° (vertical surface),

(4) $$Nu_{L}=\frac{4}{3}{\left(\frac{Gr_{L}}{4}\right)}^{1/4}g(Pr)$$

*g*(Pr) is the dimensionless temperature gradient for free convection on a vertical flat plate, and

(5) $$Gr_{L}=\frac{g\beta (T_{f})(T_{S}-T_{\infty })L^{3}}{{\nu_{g}}^{2}}$$

(6) $$Ra_{L}=\frac{g\beta (T_{S}-T_{\infty })L^{3}}{\nu_{g}\alpha }$$

The characteristic length *L* is determined by the area and perimeter of surface, that is *L* = *A*_S_*/P*, where *A*_S_ is the surface area and *P* is the surface perimeter. *β* is the coefficient of cubic expansion,

(7) $$\beta \left(T_{f}\right)=-{\left(\partial \rho /\partial T_{f}\right)}_{P}/\rho =P/\rho RT_{f}^{2}=1/T_{f}$$

where the film temperature *T_f_* = (*T_s_* + *T_∞_*)/2, and *T_f_* is used to calculate all the properties of fuel.

The thermal conductivity in gas is [20],

(8) $$k_{g}\left(T_{f}\right)=1.5207\times 10^{-11}T_{f}^{3}-4.8574\times 10^{-8}T_{f}^{2}+1.0184\times 10^{-4}T_{f}-3.9333\times 10^{-4}$$

The kinetic viscosity in gas is [20],

(9) $$\nu_{g}\left(T_{f}\right)=-1.1555\times 10^{-14}T_{f}^{3}+9.5728\times 10^{-11}T_{f}^{2}+3.7604\times 10^{-8}T_{f}-3.4484\times 10^{-6}$$

The heat transfer coefficient can be calculated as,

(10) $$h=\frac{Nu_{L}L}{k_{g}}$$

Therefore, the convective heat flux is

(11) $$q_{conv}^{\prime \prime }=h(T_{s}-T_{\infty })$$

**Figure 14:**
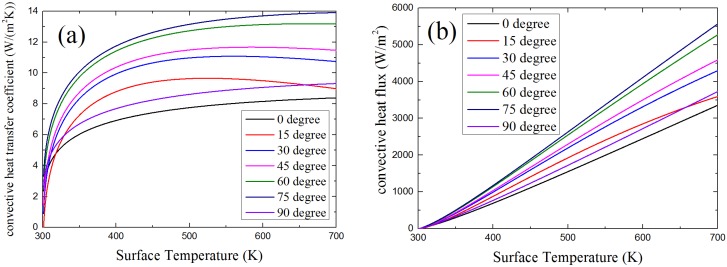
Relationship between solid surface temperature and: (**a**) convective heat transfer coefficient; (**b**) convective heat flux.

According to Equations (2)–(11), the heat transfer coefficient and the convective heat flux is calculated. The following two figures in Figure 14 show the theoretical result of relationship between convective heat transfer coefficient/convective heat flux and solid surface temperature at different orientations. As shown in the figures, except the angle of 90°, the convective heat transfer coefficient and the convective heat flux increase with orientation angle from 0° to 75° at the same surface temperature.

**Figure 15:**
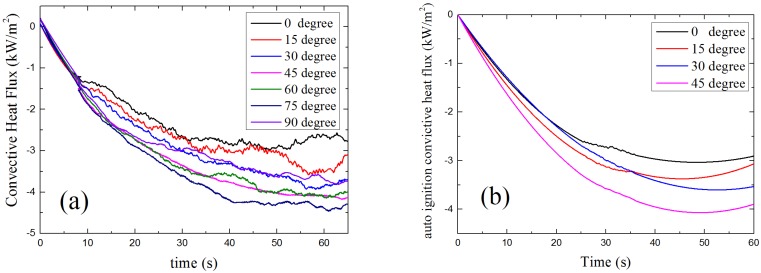
Model results of convective heat flux *vs.* time: (**a**) piloted ignition (**b**) auto-ignition.

Figure 15 shows the results of convective heat flux changes with time at a different α calculated by the FDS simulation model. The convective heat flux gets larger when α increases. With the change of orientation, the way pyrolysis gas and ambient air flow changed, the smaller α is, the smaller convective heat flux is. The profiles of calculated results of FDS model shown in Figure 14 have the same trends with theoretical analysis results.

#### 4.3.3. Net Heat Flux

**Figure 16:**
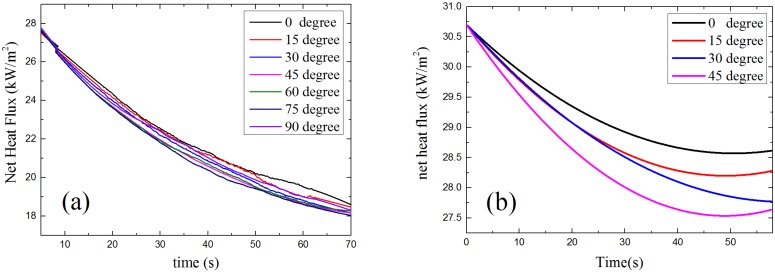
Model results: net heat flux *vs.* time: (**a**) piloted ignition (**b**) auto-ignition.

The net heat flux at sample surface is the combination of radiation and convective heat flux, which is the total heat flux that the sample surface gains from outside. The relationship between net heat flux, time and α is presented in Figure 16. As is shown, the net heat flux decreases when α becomes larger, which means with the increasing of sample orientation angle α, the sample surface gets less energy from the ambient environment. This makes the sample harder to ignite as α increases.

### 4.4. Surface Temperature

**Figure 17:**
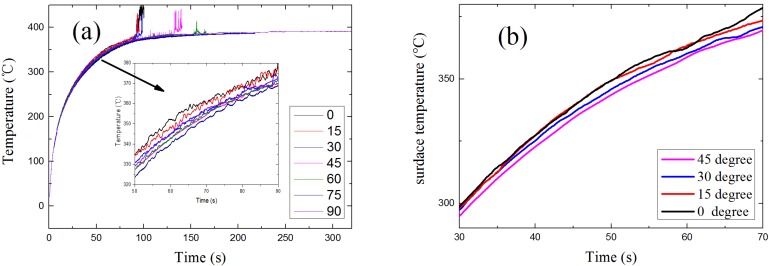
Model results: surface temperature *vs.* time: (**a**) piloted ignition; (**b**) auto-ignition.

As a result of the decreasing net incident heat flux at the sample surface, the sample surface temperature decreases with the rising of α, as shown in Figure 17. The pyrolysis reaction rate in solid phase can be calculated from Equation (12).

(12) $$S_{v}=-A_{solid}\cdot exp\left(-E_{solid}/RT_{solid}\right)$$

Lower surface temperatures results in slower pyrolysis reaction rate in solid and lower fuel concentration in gas. The reaction rate in gas phase can be calculated from Equation (13).

(13) $$S_{g}=-A_{gas}\cdot Y_{fuel}\cdot Y_{oxygen}\cdot exp\left(-E_{gas}/RT_{gas}\right)$$

As a result of lower surface temperature, *Y_fuel_* and *T_gas_* become lower. Thus, the reaction rate in gas *S_g_* is slower. Therefore, without spark igniter, lower surface temperatures make the auto-ignition harder to occur, which results in an increase of the ignition time of auto-ignition as α increases. For the piloted ignition conditions, the concentration of combustible gas at igniter place also plays an important role in the ignition of solid fuels.

### 4.5. Fuel Concentration

**Figure 18:**
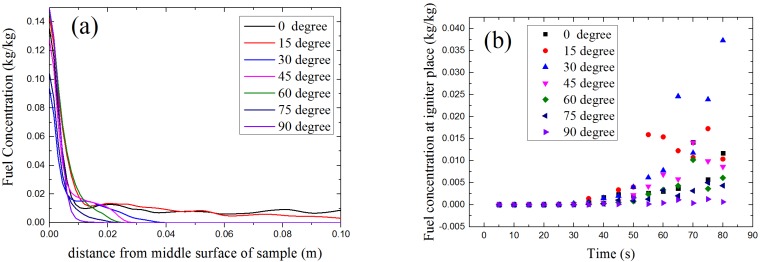
Model results: Fuel concentration *vs.* distance from sample middle surface: (**a**) Fuel concentration of middle line above sample surface at 70 s; (**b**) Fuel concentration at igniter place from 0 to 80 s.

For piloted ignition situations, Figure 18a shows fuel concentration of middle line above sample surface at 70 s, Figure 18b shows the fuel concentration at igniter place from 0 to 80 s. Like the visual inspection, these two figures reveals that the fuel layer become thinner and the fuel concentration is easier to reach 0 with the increasing α. For piloted ignition, the fuel concentration at igniter determines the ignition time. Seen from the calculation data shown in Figure 18b, the concentration of combustible volatiles at the igniter place increases first as α increases from 0° to 30°, than decreases with α from 30° to 90°. This is the key factor causes the ignition time has a U-shape. Although the concentration of combustible volatiles has the same rule with auto-ignition, the surface temperature plays the dominate role in ignition of solid fuels without igniter.

### 4.6. Discussion

Many former researchers studied the vertical and horizontal placed samples, and concluded that without a badly placed igniter, ignition occurs with difficulty in the vertical case. Similar results are observed in this work. In general, with the increase of orientation angle α, the pyrolysis gas layer become thinner as in Figure 18a, so the attenuation of radiant heat flux by volatiles gas layer is less. The incident radiant heat flux at sample surface is increasing with α under the same condition. However, the convective heat flux loss from sample surface to the ambient is also increasing with orientation angle, and the change of convective heat flux is larger than radiant heat flux between each orientation angle, so the total heat flux at sample surface is decreasing with α. That is why the surface temperature decreases with orientation angle, this leads to the ignition time become longer as α increases. In auto-ignition conditions, the total heat flux at sample surface plays the dominate role, but the gas flow of volatiles has a greater influence in piloted ignition conditions. As in Figure 18b, the higher concentration of volatile gas leads to the U-shape of ignition time.

## 5. Conclusions

Clearly, the sample orientation has a great influence on both piloted and auto-ignition of the material poly(methyl methacrylate), which has been observed experimentally and numerically in this study. Ignition times and mass flux at ignition have been measured, and by using the CFD model and theoretical analysis, the method for how orientation influence piloted ignition and auto-ignition of poly(methyl methacrylate) is discussed.

The main conclusions are as below:

(1) In piloted ignition conditions, both the ignition time and critical mass loss rate at ignition have a U-shape relationship with the sample orientation angle α. The U-shape relationships indicate there is a specific α which makes the ignition time and critical mass loss rate is at a minimum. In auto-ignition conditions, both the ignition time and critical mass loss rate at ignition decrease with sample orientation angle α, but the critical heat flux increases from 9.39 to 22.63 kW/m^2^ as α increases from 0° to 45°.
(2) Both the convictive heat flux loss and the inflow of radiant heat flux grows with sample orientation angle as α increases from 0° to 75°. As a result of the combination of the convective and radiant heat flux, the total incident heat flux at the solid surface decreases with α which makes the surface temperature of solid decrease. This makes the auto-ignition harder to occur with the increasing of the orientation angle. Because of the laminar air flow of vertical placed samples, the angle of α = 90° does not obey the same rule.
(3) Influenced by the increasing incident heat flux at sample surface and different gas flow of volatiles, the fuel concentration at the pilot place has a U-shape and results to the U-shape profile of ignition time and critical mass loss rate of piloted ignition.
